# Design and construction of a fast synthetic modified vaccinia virus Ankara reverse genetics system for advancing vaccine development

**DOI:** 10.3389/fmicb.2025.1572706

**Published:** 2025-04-25

**Authors:** Zhiqiang Gao, Busen Wang, Tianyu Liu, Zhenghao Zhao, Jinghan Xu, Xiaofan Zhao, Zhe Zhang, Zuyuan Jia, Yilong Yang, Shipo Wu, Wei Chen, Lihua Hou

**Affiliations:** Laboratory of Advanced Biotechnology, Beijing Institute of Biotechnology, Beijing, China

**Keywords:** modified vaccinia virus Ankara (MVA), reverse genetic system, rescue, viral vector vaccine, viral genomes

## Abstract

The modified vaccinia virus Ankara (MVA) is approved for use as a smallpox and monkeypox virus vaccine and was also designed as a popular recombinant viral vector for vaccine development and gene therapy. However, the extensive genomes of poxviruses present a significant challenge for the development of recombinant viral vaccines; therefore, it is essential to establish a user-friendly *in vitro* reverse genetic system. We systematically assembled the 180-kb MVA genome into a five-plasmid system, facilitating one-step packaging of the MVA virus. The MVA rescued using this system exhibited similar virological characteristics, including host cell tropism, growth kinetics, plaque size, and viral particles, comparable to those of wild-type MVA. Immunization with rescued MVA intramuscularly or subcutaneously triggered robust-specific immune responses and conferred protection against lethal attacks by the ectromelia virus in mice. We also developed a recombinant MVA-Luc-eGFP virus, which served as a tool for screening antiviral compounds against poxviruses. The synthetic MVA system efficiently generates recombinant vaccines with robust immune responses. These findings provide a novel and fast method for engineering large viral genomes with more specialized structures and lay a foundation for the advancement of more rapid and effective viral vector vaccines.

## 1 Introduction

The modified vaccinia virus Ankara (MVA) is used as a third-generation smallpox vaccine because of its remarkable safety and effective immune response (Blanchard et al., 1998; Hochstein-Mintzel et al., 1972; McCurdy et al., 2004; Pittman et al., 2019). It was derived from over 570 successive passages of the replicative vaccinia virus (VACV) strain Ankara in chicken embryo fibroblast cells (Hochstein-Mintzel et al., 1975; Mayr et al., 1978), and 12% of its genome are lost through continuous passages, causing replication defects when used in humans. Owing to the monkeypox virus (MPXV) pandemic, MVA was developed as a promising MPXV vaccine and approved for use by the US Food and Drug Administration (USFDA) in 2019 (Rao et al., 2022) and European Medicines Agency (EMA) in 2022. In addition, because of its large capacity for inserting foreign genes and safe replication within cells, MVA is also used as a popular recombinant poxvirus vaccine vector, offering significant promise for the development of vaccines against viral diseases, including respiratory syncytial virus (Jordan et al., 2024), Middle East respiratory syndrome (Koch et al., 2020), and coronavirus disease 2019 (Pérez et al., 2023; Wussow et al., 2023). Notably, the MVA-vector Ebola vaccine, MVA-BN-Filo, elicits a robust immune response when administered in combination with Ad26.ZEBOV and has also received marketing authorization from the EMA (Afolabi et al., 2022; Yin et al., 2024).

Despite the widespread use of MVA in the life sciences and biomedical fields, it is not easy to modify the MVA genome because of its huge 180-kilobase (kb) genome. Traditionally, the standard method for inserting genes involves transferring plasmids into mammalian or avian cells to enable homologous recombination with MVA. Screening for recombinant viruses requires multiple rounds of plaque purification (Mackett et al., 1982). Editing multiple regions of the MVA genome requires numerous genomic modifications, rendering the procedure labor-intensive and intricate.

The development of an MVA reverse genetic system through DNA synthesis and genome assembly techniques facilitates the manipulation of mega-genomes *in vitro*. The first reverse genetics system for poxviruses was established by Domi and Moss (2002) through the integration of a bacterial artificial chromosome utilizing the circularization of head-to-tail concatemers of VACV. In Cottingham et al. (2008) successfully generated four full-length rescuable BAC-MVA clones using a methodology analogous to that developed by Domi. In Chiuppesi et al. (2020) divided the genome of the modified vaccinia Ankara (MVA) virus into three subgenomic fragments and rescued the MVA using two different fowlpox virus (FPV) (). Nevertheless, while the previously mentioned viral vector systems can achieve virus rescue, the plasmids harboring the viral genomes remain substantial in size, which complicates the process of *in vitro* genomic manipulation.

In this study, we constructed a novel MVA reverse genetics system consisting of five plasmids covering the full viral genome from chemically synthesized DNA, which is convenient for genome editing *in vitro* and is suitable for the simultaneous insertion of multiple exogenous proteins. The system showed high efficiency in virus packing, and the rescued virus exhibited virological characteristics and immunology comparable to those of MVA-WT, demonstrating that it is a promising vehicle for the development of MVA-associated vaccines.

## 2 Materials and methods

### 2.1 Viruses and cell culture

MVA-WT (VR1508) was procured from the American Type Culture Collection, FPV was sourced from the National Center for Veterinary Culture Collection, and all virus stocks were stored in aliquots at a temperature below –80°C. DF-1 cells (BNCC100311) were purchased from BeNa Culture Collection, BSC40 cell (YS2791) was acquired from Shanghai Yaji Biotechnology Co., Ltd., and BHK-21, A549, Vero E6, and HEK 293T cells were maintained in our laboratory. The cells were cultured at 37°C in a 5% CO_2_ incubator and maintained in Dulbecco’s Modified Eagle Medium (DMEM) (Thermo Scientific, USA), supplemented with 10% fetal bovine serum (Thermo Scientific, USA), 100 units/mL of penicillin, and 100 μg/mL of streptomycin.

### 2.2 Bacteria and plasmids

The transformation-associated recombination (TAR) cloning vector, BACYAC (pYES1L), was obtained from Thermo Fisher Scientific and was characterized by its resistance to spectinomycin and the presence of a tryptophan (TRP) biosynthesis gene. The plasmid for gibson assembly (PET) vector was maintained in the laboratory. Saccharomyces cerevisiae strain VL6-48N (genotype: MATα, his3-Δ200, trp1-Δ1, ura3-Δ1, lys2, ade2-101, met14, cir0) was procured from Shanghai Zeye Biotechnology Co., Lt. for use in all TAR-related experiments. This strain was cultured in a synthetic defined medium supplemented with TRP.

### 2.3 Synthesis of the MVA genome

**FIGURE 1:**
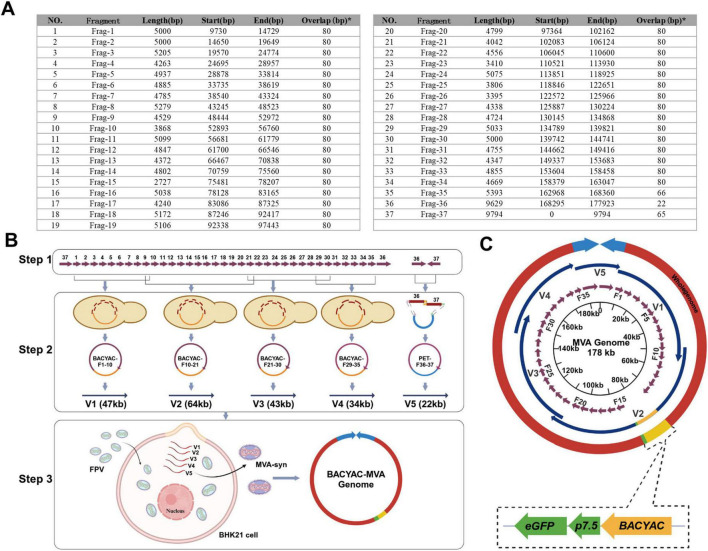
Assembly of MVA-syn genome and rescue of recombinant MVA virus. **(A)** The full chemical synthesis fragment length and the start and end points within the MVA genome. *The overlap sequence between the fragment and its next fragment. **(B)** The flowchart delineates the MVA genome assembly process. Step one, the process commences with the comprehensive chemical synthesis of fragments F1 through F37. Step two, the process involves the combination of fragments F1–F10, F10–F21, F21–F30, F29–F35, and F36–F37 to assemble fragments of 47, 64, 43, 34, and 22 kb, respectively. Step 3, the FPV infects BHK-21 cells for 1 h, and the MVA-syn is subsequently rescued through co-transfection with five enzymatically digested and linearized plasmids. **(C)** A circular graphical map of the genome of MVA-syn. Within the innermost circle, purple arrows indicate the 37 chemically synthesized fragments, labeled F1–F37. The second layer features dark blue arrows that represent genomic segments designated as V1 through V5. Yellow segments denote the BACYAC plasmid and green segments represent the *eGFP* gene. The entire genome is depicted by the outer red circle and the light blue arrows illustrate the two ITRs.

The MVA genome (U94848.1) was segmented into 37 distinct DNA fragments, with each fragment varying in length from 5 kb to 10 kb. All fragments of F1-34 share a homologous region of 80 bp with each adjacent fragment, F35 shares a 66 bp homology region with F36, F36 shares a 22 bp homology region with F37, and F37 shares a 65 bp homology region with F1 (Figure 1A). The synthesis of the F1-20 and F21-37 fragments was commissioned to GenScript and SinoGenoMax, respectively. Fragments F1-20 were produced without the addition of cleavage sites at their termini, and the *eGFP* gene was inserted into the thymidine kinase gene of F15. The synthesized fragments F1-20 served as templates for the PCR amplification and the primers are listed in Supplementary Table 1. PCR products were purified according to the protocol outlined in the MiniBEST Agarose Gel DNA Extraction Kit Ver.4.0 (Takara, 9762). Fragments F21-35 were synthesized with AsisI cleavage sites at both ends, and fragments F36-37 included both AsisI and XmaI cleavage sites (Supplementary Table 2). The digest products were purified in accordance with the protocol outlined in the MiniBEST DNA Fragment Purification Kit Version 4.0 (Takara, 9761). The 37 chemically synthesized DNA fragments were allocated to five plasmids for subsequent assembly.

### 2.4 Transformation-associated recombination

The transformation mixture was prepared by combining 100 ng of linearized BACYAC vector with 200 ng of each DNA fragment and 500 ng of stitching oligonucleotides, as detailed in Supplementary Table 3. These stitching oligonucleotides contain 80 bp homologous region at the termini of each pair of fragments to be joined. This homologous region facilitates the splicing of two DNA fragments that lack inherent homology, DeMarini et al. (2001) while also incorporating an AsisI or AscI enzyme cleavage site at the junction where the homologous region anneals. VL6-48N competent cells were thawed to ambient temperature and gently inverted 5–8 times to achieve homogeneous suspension. Subsequently, 100 μL of VL6-48N cell suspension was aliquoted into the DNA mixture with brief tube tapping to ensure complete dissolution. Following addition of 600 μL PEG/LiAc solution, the mixture was homogenized by gentle inversion. The reaction system was supplemented with 35.5 μL DMSO and subjected to thermal shock at 42°C for 20 min with intermittent mixing at 5-min intervals. Post-incubation, the samples were centrifuged at 400 *g* for 5 min at room temperature. The supernatant was carefully aspirated and the pellet was resuspended in 1 mL sterile 0.9% NaCl solution. Finally, 100 μL aliquots of the cell suspension were plated onto SD-Trp selective medium and incubated at 30°C for 72 h under static conditions. PCR was employed to identify positive VL6-48N yeast colonies, from which recombinant plasmid was then extracted. Combine 100 μL DH10B competent cells with 5 μL plasmid DNA, thaw cells on ice, and incubate for 15 min. Transfer the mixture to a pre-chilled cuvette without air bubbles and apply an electric pulse (1.8 kV, 25 μF, 200 Ω) using a Bio-Rad Gene Pulser. Add 1 mL of 37°C SOC medium immediately, transfer to a 15 mL tube, and incubate at 37°C and 220 rpm for 1–1.5 h. Spread 100–200 μL of the culture on LB plates with spectinomycin and incubate at 37°C for 16–20 h. Select 8–10 colonies for colony PCR using Taq polymerase and confirm positive results with a restriction digest and sanger sequencing.

### 2.5 Gibson assembly

Plasmid V5 incorporated F36 and F37, which were acquired through the transformation of *E. coli* via Gibson assembly. The terminal region of the synthesized F36 extends the DNA sequence by 11 base pairs, facilitating the creation of a 22 bp palindromic sequence at the terminus of the F36. This palindromic sequence enables the F36 to utilize the 22 base pair homologous region for recombination with the F37, resulting in the formation of an inverted terminal repeat (ITR) head-to-head dimer structure. The linearised vector verified (50 ng) by agarose gel electrophoresis was mixed with purified DNA fragments in a 1:2 molar ratio in a sterile PCR tube. Subsequently, 10 μL of Gibson Assembly Mix (NEB, E2621L) was added, and sterile nuclease-free water was used to adjust the total volume to 20 μL, and the reaction was incubated at a constant temperature of 50°C for 30 min, take 2 μL of the reaction product and 50 μL of TOP10 (Tiangen Biotech, CB104) chemosensory cells incubated in an ice bath for 30 min, 42°C water bath heat excitation for 90 s and immediately after the ice bath for 2 min, add 500 μL of pre-warmed LB liquid medium, and the mixture was shaken at 220 rpm at 37°C for 45 min. 100 μL of the transformation solution was evenly spread on the LB plate containing the kanamycin antibiotics, and then inverted at 37°C for 12–16 h. Single colonies with a diameter of 1–2 mm were picked for colony PCR verification and confirm positive results with a restriction digest and sanger sequencing.

### 2.6 Transfection and infection assays

Five vectors were isolated from *E. coli* using methods developed for large plasmids (Thermo Fisher Scientific, K210017). BHK-21 cells were seeded in 6-well plates (4 × 105 cells/well) and cultured overnight in DMEM with 10% FBS to achieve 80%–90% confluency. Cells were infected with fowlpox virus (FPV) at an MOI of 0.5 in serum-free DMEM for 2 h, followed by replacement with fresh complete medium. Concurrently, the MVA backbone plasmids (V1–V5) were linearized using restriction enzymes. A transfection mixture containing 1 μg of each plasmid and Turbofect reagent (6 μL) in serum-free DMEM was incubated for 20 min and added to FPV-infected cells. Post-transfection, cytopathic effects (CPE) were monitored daily until full manifestation (48–72 h). Cells were harvested via freeze-thaw cycles (−80°C), and 2 mL lysate was passaged onto fresh BHK-21 monolayers for amplification. Recombinant MVA viruses (designated P0) were successively propagated (P1/P2), with intracellular eGFP fluorescence confirmed by microscopy at 24 h post-infection.

### 2.7 Identification of the MVA-syn and MVA-HA genome

Five synthetic MVA poxvirus monoclones (MVA-syn1–5) were isolated using plaque purification, and the genomes of MVA-syn1–5, MVA-WT, MVA-HA and FPV were extracted using the Viral Genome DNA/RNA Rapid Extraction Kit (Tengen Biochemistry Science and Technology [Beijing] Co., Ltd., DP307). Subsequently, the genomes were identified using PCR (Supplementary Table 4), and processed for Illumina sequencing of MVA-syn4 following the standard protocol of the Sequencing Platform of GENEWIZ (Suzhou, China).

### 2.8 Titre measurement of virus

MVA was quantified in BHK-21 cells by measuring infectious units (IFUs). Immunostaining of viral plaques formed following cell infection was conducted using Rabbit Polyclonal Antibodies specific to VACV (ab35219, Abcam). The stained plaques were subsequently counted under a microscope to determine the viral titres.

### 2.9 Host cell tropism and growth curve assay

The MVA-syn1, MVA-syn4, MVA-syn5, and MVA-WT strains were infected into BHK-21, DF-1, A549, HEK293T, Vero-E6, and BSC40 cell lines at a multiplicity of infection (MOI) of 0.01. The cultures were maintained in 2% DMEM maintenance medium. Viral supernatants were collected at 1, 12, 24, and 72 h post-infection with three replicates for each time point. Viral titres were quantified in BHK-21 cells.

### 2.10 Plaque size measurements

MVA-syn1, MVA-syn4, MVA-syn5, and MVA-WT were used to infect a monolayer of BHK-21 cells in a 6-well plate at an MOI of 0.001. The culture medium was removed after infection, and the cells were rinsed thrice with phosphate-buffered saline (PBS). The monolayer was then overlaid with cryo agar and incubated at 37°C for 72 h. Subsequently, the medium was discarded, and the cells were fixed with 4% paraformaldehyde histiocyte fixative at room temperature for 12 h. The cells were then stained with crystal violet, and the plates were scanned using a Celigo full-field cell analyser (Nexcelom). The size of the phagocytic spots within the specified area was quantified using the ImageJ software.

### 2.11 Bio-transmission electron microscopy

BHK-21 cells were infected at an MOI of 1 for 24 h. The cells were fixed with gluta fixative (2.5% for electron microscopy) and collected for observation using Bio-transmission electron microscopy (TEM). MVA poxvirus was purified using 25% sucrose, and viruses were fixed using gluta fixative (for electron microscopy, 2.5%) for electron microscopy.

### 2.12 Antigen insertion

A PET-mH5-HA plasmid was constructed using a PET vector. The restriction enzyme PacI digested the V3 plasmid and purified the digested product using the ethanol precipitation (Takara, 9094) method, PCR amplified the HA protein expression frame, and the above two fragments were spliced using Gibson. We replaced the eGFP of F15 with Luc-P2A-eGFP, prepared a V2 plasmid by TAR recombination, and packaged the recombinant MVA-HA and MVA-Luc-eGFP viruses using the MVA reverse genetics system.

### 2.13 Luciferase assay

A luciferase reporter gene assay was conducted using a Luciferase Reporter Gene Assay Kit (DD1204-03, Vazyme) to quantify luciferase activity. Following the removal of 100 μL of culture medium, the luciferase assay solution was introduced. The luminescence was subsequently measured using the GloMax^®^ Navigator System (GM2000, Promega).

### 2.14 Evaluation of antiviral compounds targeting poxvirus infections

Compounds were serially diluted to a concentration of 200 nM/mL and added to BHK-21 cells 6 h after MVA-Luc-eGFP infection, with 1% dimethyl sulfoxide (DMSO) as control. The cell lysates were harvested 24 h after treatment, and the inhibition rate of the compounds on the luciferase signal was determined relative to that of the control group treated with DMSO.

### 2.15 Western blotting

BHK-21 cells were inoculated into six-well cell culture plates and transiently infected with equal amounts of MVA-HA at an MOI of 1. Following 24- and 48-h incubation periods, the culture supernatant was discarded and the lysed cells were washed with 150 μL of Radioimmunoprecipitation Assay Lysis and Extraction Buffer (Thermo Scientific, USA). The resulting lysates were centrifuged and mixed with the LDS buffer (Thermo Fisher Scientific). The samples were then subjected to electrophoresis on a 4%–20% polyacrylamide gel (GenScript, China) and transferred to a nitrocellulose membrane (Bio-Rad, China). To block non-specific binding, the membranes were treated with Tris-HCl containing 0.1% Tween 20 and 5% skim milk for approximately 1 h. The membranes were then incubated with a rabbit monoclonal antibody against haemagglutinin (HA) (86001-RM01, SinoBiological) at a dilution of 1:2000. After washing, the primary antibody was detected using goat anti-rabbit IgG conjugated with horseradish peroxidase (HRP) (Cell Signaling Technology). Following another washing step, the membranes were developed using an ECL substrate (Thermo Scientific, USA), and images were captured using the ChemiScope system (Clinx Science, China).

### 2.16 Animal experiments

All animal experiments were performed in accordance with the guidelines of the Institutional Laboratory Animal Welfare and Ethics Committee. In total, 42–56-day-old BALB/c mice were purchased from Vital River Laboratories (Beijing, China) and used for all animal immunization experiments. All immunized samples used in this study were purified using 25% sucrose, 5 × 10^7^ IFUs in the high-dose group and 5 × 10^6^ of MVA-syn4, MVA-WT, and MVA-HA in the low-dose group for intramuscular and dorsal cervical subcutaneous immunizations using two-dose regimen on day 0 and day 21, respectively. Serum was collected using the retro-orbital hemorrhage method for humoral immunological analysis at week 3 after primary immunization and 1 week after booster immunization. For the analysis of cellular immunity in mice immunized with MVA-HA, splenocytes were harvested 2 weeks after booster immunization. The cells were isolated using standard procedures following humane euthanasia. On day 56, ectromelia virus (ECTV) challenges were evaluated in BALB/c mice immunized with MVA-syn4 or MVA-WT by intraperitoneal injection of 1,000 IFUs. The mice were subsequently monitored for survival, changes in body weight, and occurrence of adverse reactions. In addition, MVA-Luc was administered to BALB/c mice via intramuscular and subcutaneous injections at doses of 5 × 10^7^ and 5 × 10^6^ IFUs, respectively. *In vivo* luminescence imaging was performed 3 days after infection using an IVIS Spectrum *In Vivo* Imaging System. The mice were intraperitoneally injected with 250 mg of Perkin Elmer IVISbrite D-Luciferin Potassium Salt Bioluminescent Substrate (IVISbrite) dissolved in PBS and exposed for 5 min.

### 2.17 Enzyme-linked immunosorbent assay

The purified MVA and VACV were inactivated by sonication in a water bath for 3 min. Enzyme-linked immunosorbent assay plates (Corning, 9018) were coated with 1 × 10^7^ IFU/mL dilutions of virus inactivation products, HA protein 2 mg/mL, and incubated overnight at 4°C. The plate were washed thrice with 1 × PBS with Tween 20 and then blocked with 2% bovine serum albumin in PBS for 1 h at 37°C. Serum samples were heat inactivated at 56°C for 30 min and were diluted in assay buffer; then, 50 μL diluted samples were added to the respective wells and incubated for 1 h at 37°C. After washing as described above, enzyme-linked goat anti-mouse IgG-HRP (Santa Cruz, sc-200) was incubated for 1 h at 37°C. After washing, it was incubated with 100 μL TMB (Solarbio, PR1200) for 6 min, 50 μL of TMB stop solution (SolarbioM, C1058) was added, and plate OD values were read at 450 nm and 630 nm. All data were analyzed using the GraphPad Prism software.

### 2.18 Neutralization assay

Poxvirus-neutralizing antibodies were detected using MVA-Luc and VACV-Luc. In the serum neutralization assay, samples were heat inactivated at 56°C for 60 min, and the virus was diluted and mixed with serial dilutions of the samples in a 96-well plate. After incubation at 37°C for 1 h, the cells were inoculated with cells. After 24 h, the luciferase activity of the samples was measured using a Bright-Lite luciferase assay system (Vazyme, DD1204-03). Using the Reed–Muench method, the neutralizing titre 50% neutralization potency was calculated as the reciprocal of the dilution at which the luciferase activity reached half of the negative control.

### 2.19 Enzyme-linked immunosorbent spot analysis

Enzyme-linked immunosorbent spot plates were initially coated with 5 μg/mL of anti-mouse interferon (IFN)-γ antibody and incubated overnight at 4°C. Subsequently, the plates were rinsed five times with PBS and blocked with complete Roswell Park Memorial Institute 1640 for 2 h at room temperature. Following this, 100 μL of a splenocyte suspension, containing 2 × 10^6^ cells/mL and a pool of HA peptides at a concentration of 1 μg/mL, was added to each well. A negative control devoid of peptides was included in all the experimental setups. The plates were then incubated at 37°C with 5% CO_2_ for 18–24 h. After incubation, the plates were washed, and biotin-labeled anti-mouse IFN-γ, at a concentration of 1 μg/mL, was added and allowed to incubate for 2 h at room temperature. After three washes, streptavidin-HRP was added to each well and incubated for 1 h. The plates were then washed five times with PBS, and a color development reaction was initiated using 3,3′,5,5′-tetramethylbenzidine as the substrate. Upon completion of the color development, the membrane was left to dry overnight in the dark. The spot samples were subsequently counted using an AT-Spot 3200 (SinSage Technology, Beijing, China), and the results are expressed as the number of spot-forming cells.

### 2.20 Statistical analysis

The analysis was performed with GraphPad Prism v.7.00. Unpaired *t*-tests were conducted to compare differences between two experimental groups. One-way ANOVA with Tukey’s multiple comparisons tests were applied to compare more than two experimental groups. A *p*-value of less than 0.05 was considered statistically significant, with significance levels denoted as follows: *p < 0.05; **p < 0.01; ***p < 0.001; and ns, for not significant. The antibody titer data were log transformed before analysis. The error bars throughout all the figures represent one standard deviation.

## 3 Results

### 3.1 Synthesize and assembly of MVA genome

The MVA genome (U94848.1) obtained from GenBank was systematically segmented into an average of 37 fragments (Figure 1A). Among these, F1-35 covered an average length of 5 kb, and F36 and F37 corresponded to the left and right inverted terminal repeats (L-ITR and R-ITR, respectively), each measuring 9.8 kb in length. The purified results of F1-37 are presented in Supplementary Figure 1. All 37 fragments were prepared, and the genome was subsequently assembled through TAR method and Gibson assembly. The 37 DNA fragments were allocated to five plasmids. As shown in Figures 1B, C, the TAR method was used to construct V1–V4 plasmids, each successfully cloned in a single event. The process involved linearized plasmids and DNA fragments, as well as stitching oligonucleotides, BACYAC-F1-10 (V1), BACYAC-F21-30 (V3), BACYAC-F29-35 (V4) fragments with *AsisI* and *Asc*I cleavage sites added at both ends by stitching oligonucleotides. Similarly, BACYAC-F10-21 (V2) contains fragments F10-F21, where an *AsisI* site was introduced at the junctions of F15-BACYAC for the insertion of exogenous genes, and an *Asc*I site was introduced at the junction of F10-21. Furthermore, the PET plasmid introduces enzymatic sites to the PCR product ends by using primers with homology to F36 and F37, see Supplementary Table 1 for primer details, the *AsisI* and *Asc*I cleavage sites were introduced at the PET-F36 and F37-PET junctions. And the accurate positive clone was successfully acquired through a single application of the Gibson assembly.

**FIGURE 2:**
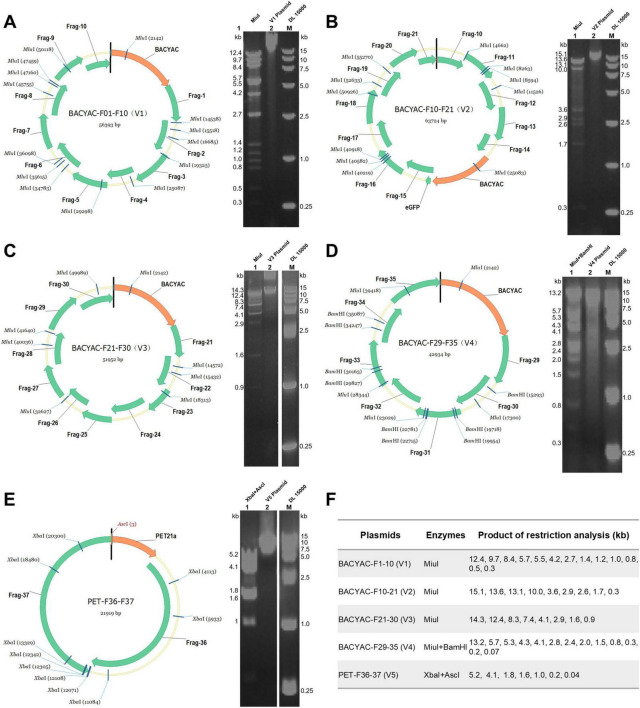
Restriction enzyme analysis of the synthetic V1–V5 plasmids. **(A)** The mapping and restriction enzyme profiles of the V1 plasmid. **(B)** The mapping and restriction enzyme profiles of the V2 plasmid. **(C)** The mapping and restriction enzyme profiles of the V3 plasmid. **(D)** The mapping and restriction enzyme profiles of the V4 plasmid, DNA fragments smaller than 0.3 kb are not labeled because the bands are not obvious. **(E)** The mapping and restriction enzyme profiles of the V5 plasmid. **(F)** The size of the digested products of V1–V5 plasmids.

The mapping of the V1–V5 plasmids is shown in Figure 2. V1–V3 plasmids isolated from *E. coli* was digested with MiuI (Figures 2A–C), V4 plasmid was digested with MiuI and BamHI (Figure 2D), V5 plasmid was digested with AscI and XbaI (Figure 2E), the results demonstrated that the V1–V5 plasmids displayed correct electrophoresis profiles, as expected (Figure 2F). And the accurate sequencing of the five plasmids was validated through Sanger sequencing. Linearized plasmids V1–V5 were transfected into FPV-infected BHK-21 cells. To rescue synthetic MVA poxviruses, the plasmids BACYAC-F1-10 (V1), BACYAC-F21-30 (V3), BACYAC-F29-35 (V4), along with PET-F36-37 (V5) could be removed from the backbone plasmid by AscI and AsisI enzymes, exposing the terminal homologous region, and BACYAC-F10-21 (V2) was cleaved by AscI to expose the terminal homologous regions of F10 and F21. Ultimately, all linearized plasmids were co-transfected into FPV-infected BHK21 cells, leading to the packaging of recombinant MVA (Figure 1B). This study involved the strains MVA-syn, MVA-Luc-eGFP, and MVA-HA, each of which was successfully acquired through a single rescue event using the previously described standard method. Therefore, we propose that this method exhibits a high level of rescue efficiency.

### 3.2 Characterization of recombinant MVA-syn poxviruses

**FIGURE 3:**
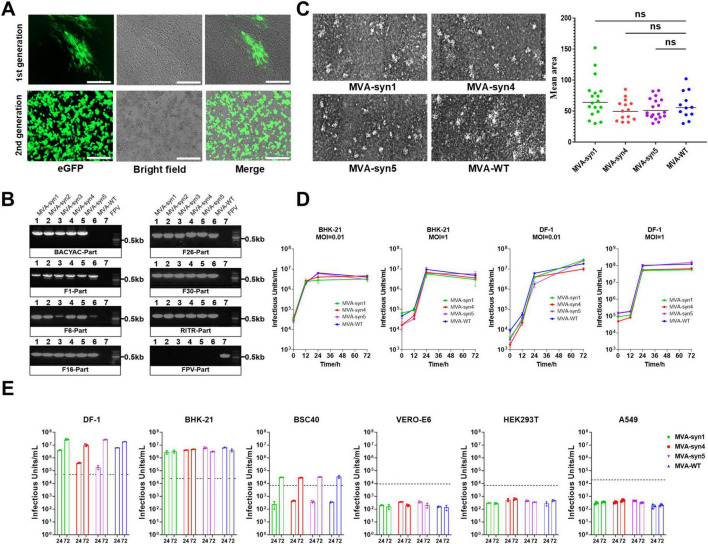
Characterization and properties of MVA-syn. **(A)** BHK-21 cells were infected with both the first and second generations of MVA-syn, and images were captured 24 h post-infection. **(B)** Five MVA-syn clones were isolated through plaque purification, virus genome DNA was extracted, and specific primers were used to amplify part of the chemically synthesized DNA, including BACYAC plasmid, F1, F6, F16, F26, F30, RITR and FPV. Control groups consisted of cells infected with MVA-WT and FPV, respectively. **(C)** Three MVA-syn viruses were used to infect BHK-21 cells at a MOI of 0.001 to analyze viral plaque sizes. Seventy-two hours later, plaque areas were measured, and statistical analyses were performed to summarize the dimensions for plaques of each virus. **(D)** The growth kinetics were evaluated in BHK-21 and DF-1 cell lines at a MOI of 0.01 and 1. Viral samples were taken at 0, 12, 24, and 72 h post-infection, and the viral titer was measured in BHK-21 cells (*n* = 3). **(E)** Host cell tropism: Human cell lines (HEK293, A549) and others (Vero-E6, BSC-40, DF-1, BHK-21) were infected with MVA-syn or MVA-WT at a MOI of 0.01. Viral titres were measured at 24 and 72 h post-infection (*n* = 3). The dashed line indicated the viral titre of the inoculum, calculated based on an MOI of 0.01. Significance of results were evaluated by unpaired *t*-tests between two groups. *p*-value less than 0.05 was considered significant. ns., not significant.

The first- and second-generation progeny viruses, which were transmitted sequentially after packaging, were analyzed using fluorescence microscopy at 48 h post-infection of BHK-21 cells. Distinct eGFP fluorescence was observed, indicating successful infection (Figure 3A). Furthermore, second-generation viruses induced characteristic cellular lesions, whereby the infected cells lost their original morphology and became rounded and hypertrophied. This result convincingly demonstrates that transfecting cells with the pentameric plasmid system led to the production of infectious progeny. Five MVA-syn clones were isolated from BHK-21 cells via plaque purification. To characterize MVA-syn viral DNA, the viral genome was extracted following viral proliferation in BHK-21 cells. Subsequently, several genomic locations in MVA-syn- and MVA-WT-infected DNA extracts were compared using PCR. The results demonstrated that all assessed genomic loci of MVA-syn and MVA-WT produced comparable PCR results (Figure 3B). Furthermore, neither sample exhibited FPV helper viruses, suggesting that MVA-syn viral passaging and phage purification are effective in eradicating helper viruses. To ensure the genomic fidelity of MVA-syn, we first sequenced the genome of MVA-syn4 from our viral collection (see “2 Materials and methods) by Illumina sequencing. The genomic data of MVA-syn4 have been uploaded to NCBI and have been assigned the GenBank accession number PV358350. Owing to the highly repetitive ITR structures, sequencing coverage was limited to the core genomic region (positions 9,867–172,157 bp of reference U94848.1). Comparative genomic analysis and targeted Sanger sequencing confirmed sequence fidelity, with no detectable nucleotide variations relative to the expected synthetic construct five plasmids. Plaques from MVA-syn1, MVA-syn4, MVA-syn5, and MVA-WT BHK-21 cells were similar in size (Figure 3C).

### 3.3 *In vitro* growth characterization of MVA-syn poxviruses

To elucidate the replicative properties of MVA-syns, we compared their replication with that of MVA-WT across various cell lines. MVA-syns and MVA-WT exhibited limited viral replication in several cell lines (Figure 3E). Specifically, neither virus replicated in HEK293T and Vero-E6 cells, and restricted replication was observed in BSC40 cells. In contrast, all viruses demonstrated complete replication in BHK-21 and DF-1 cells. Consequently, we compared the growth kinetics of MVA-syns and MVA-WT in BHK-21 and DF-1 cells and found that both viruses exhibited similar growth patterns (Figure 3D).

### 3.4 Physical properties of MVA-syn4 poxviruses

**FIGURE 4:**
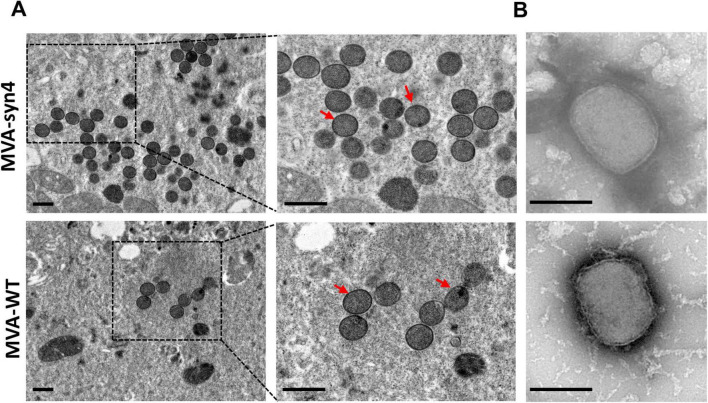
Visualization by electron micrographs of MVA-Syn4 and MVA-WT. **(A)** Electron micrographs of ultrathin sections of BHK-21 cells infected with MVA-syn4 and MVA-WT. **(B)** Electron microscope images of the purified sucrose MVA-syn4 and MVA-WT viral particles. The viral partical were indicated with arrows. Scale bars = 250 nm.

To compare the morphology of the synthesized MVA-syn4 poxviruses with that of MVA-WT, we conducted TEM analyses of BHK-21 cells infected with MVA-syn4 and MVA-WT. The two viruses exhibited similar shapes and sizes, and both possessed smooth surfaces (Figures 4A, B). TEM analysis indicated that the viral particles of both types were normal in size and structure and contained a substantial number of occlusion-derived virus particles.

### 3.5 Comparison of immunogenicity of MVA-syn4 and MVA-WT

**FIGURE 5:**
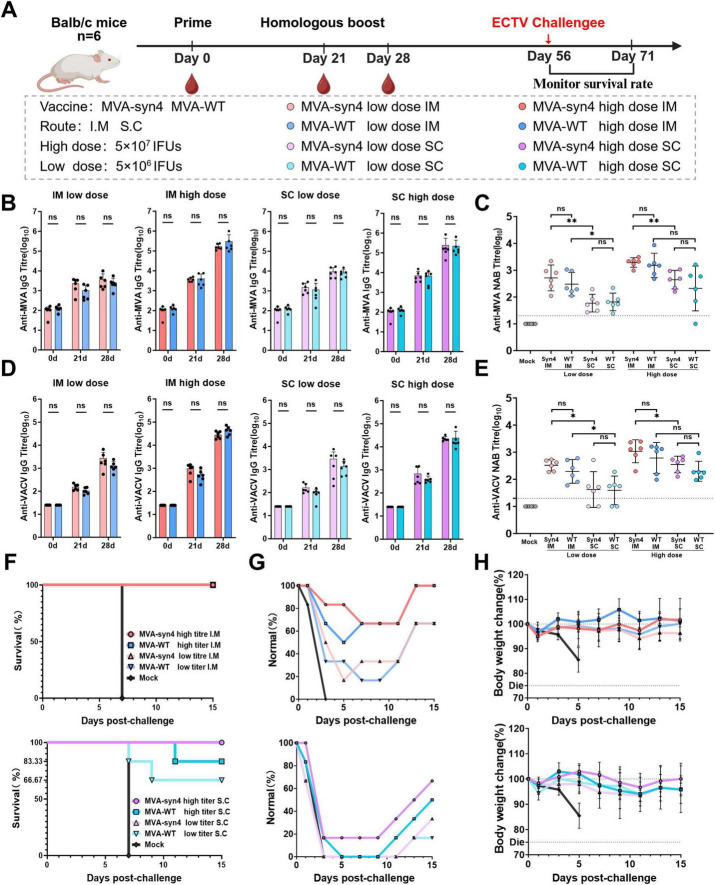
Comparison of immunogenicity between MVA-syn4 and MVA-WT. **(A)** The shcedule of immunization and challenge periods in days, immunizations were performed intramuscularly and subcutaneously, and challenge was performed by intraperitoneal injection (*n* = 6/group). **(B,C)** Anti-MVA and anti-VACV IgG titers were evaluated on days 0, 21, and 28. The crossbars indicate the geometric mean of the group, accompanied by the geometric standard deviation (*n* = 6 per group). **(D)** Anti-MVA Nab titers on day 28 was measured by recombinant MVA expressing a luciferase protein. **(E)** Anti-VACV Nab titers on day 28 was measured by recombinant VACV expressing a luciferase protein. **(F)** Survival percentages of each group during the challenge period (*n* = 6/group). **(G)** Proportion of normal mice that did not develop dull fur in each group during the challenge period. **(H)** Weight changes were monitored for 20 days post infection. Differences between groups were evaluated using one-way ANOVA with Tukey’s multiple comparison test. Significance of results were evaluated by unpaired *t*-tests between two groups. *p*-value less than 0.05 was considered significant. *p < 0.05; **p < 0.01; ns., not significant.

To assess the immunogenicity of MVA-syn4 *in vivo*, we performed a comparative analysis of the immunogenic responses elicited by MVA-syn4 and MVA-WT following two homologous immunizations administered via intramuscular and subcutaneous injections at high and low doses in BALB/c mice Figure 5A. Regardless of the route of administration, whether intramuscular or subcutaneous, the levels of binding and neutralizing antibodies against MVA induced by MVA-syn4 and MVA-WT were consistent across the high- and low-dose groups (Figures 5B, C). We assessed the cross-reactivity to the VACV virus after immunization with MVA-syn4 and MVA-WT, both of which generated binding and neutralizing antibodies against VACV across the two different injection modalities, and the concentrations of the binding and neutralizing antibodies against VACV remained consistent (Figures 5D, E). Importantly, the humoral immune response elicited by intramuscular injection was significantly more robust than that induced by subcutaneous immunization, when utilizing the same vaccine and immunization dose across different modalities.

### 3.6 Evaluation of protective efficacy against ECTV in mice

The protective efficacy of MVA-syn4 and MVA-WT immunization in mice was assessed using an ECTV lethality model, which can be conducted under biosafety level 2 conditions. Our findings indicated that, when challenged with 1,000 IFUs of ECTV 37 days after boost immunization in the intramuscular cohort, the high- and low-dose groups conferred complete protection (Figure 5F). The incidence of dull fur, a physiological phenomenon, was lower in the high-dose group than in the low-dose group (Figure 5G). Additionally, the degree of weight loss observed in the mice was less pronounced in the high-dose group than in the low-dose group, and the rate of weight recovery was more rapid in the high-dose group than in the low-dose group (Figure 5H). In the subcutaneous immunization cohort, high-dose MVA-syn provided complete protection, with 83.33% of survival in the high-dose MVA-WT group and 83.33% efficacy for MVA-syn4 and 66.67% for MVA-WT in the low-dose group. All mice in the subcutaneous immunization cohort exhibited a physiological state characterized by dull fur. Mice in the high-dose group recovered more quickly and experienced less weight loss than those in the low-dose group. Overall, the level of immunoprotection provided by intramuscular injection was superior to that provided by the subcutaneous method, and this finding was consistently observed throughout this study.

### 3.7 Construction and characterization of MVA-Luc-eGFP

**FIGURE 6:**
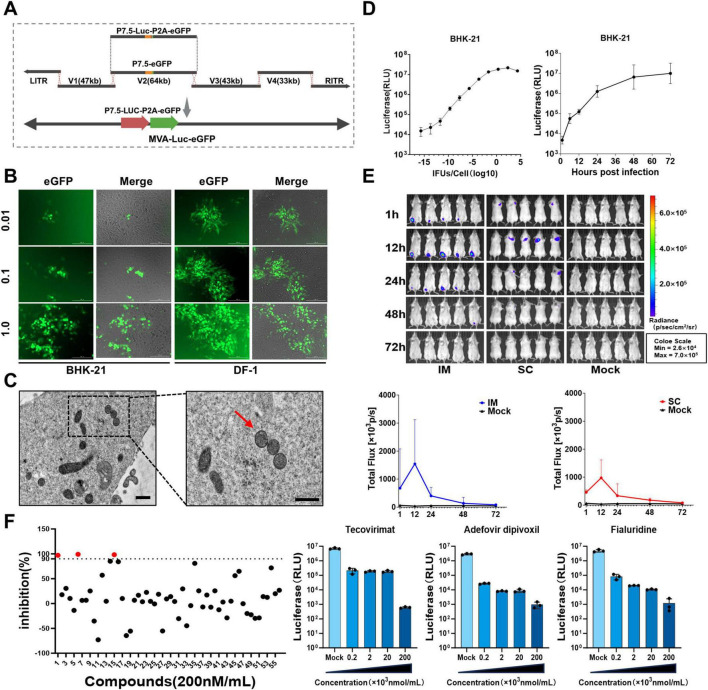
Construction, characterization and application of MVA-Luc-eGFP. **(A)** The MVA-Luc-eGFP recombinant virus was generated by substituting the *eGFP* gene in the vector V2 region with the “*Luc-P2A*-*eGFP*” construct. **(B)** BHK-21 and DF-1 cells were infected in culture for 24 h at MOI of 0.01, 0.1, 1.0, and images were captured 24 h post-infection. **(C)** Electron microscope images depict viral particles within BHK-21 cells and purified viral particles isolated using sucrose gradient centrifugation. The viral partical were indicated with arrows. Scale bars = 250 nm. **(D)** Serial dilutions of the MVA-Luc-eGFP recombinant virus were added to 2 × 10^4^ BHK-21 cells in a 96-well plate, luciferase expression was also measured at 24 h post-infection. And BHK-21 were infected at MOI of 0.1, and luciferase expression was also measured at 1, 6, 12, 24, 48, 72 h post-infection. **(E)** Live imaging of MVA-Luc-eGFP after the intramuscular and subcutaneous administration, and the *in vivo* luciferase signal intensity was measured at various time points in BALB/c mice. **(F)** Inhibition of 56 candidate compounds against MVA, red dots represent three compounds exhibiting inhibition rates exceeding 90% and dose-response effects of three inhibitors.

The V2 plasmid was reconstructed using TAR recombination, in which the *eGFP* gene was substituted with Luc-P2A-eGFP (Figure 6A). Recombinant viruses were introduced into BHK-21 and DF-1 cells at MOIs of 0.01, 0.1, and 1 (Figure 6B). Forty-eight hours post-infection, fluorescence microscopy revealed eGFP expression in all individual plate wells, with expression levels increasing proportionally with higher MOIs. TEM was used to analyze the viral particles of MVA-Luc-eGFP (Figure 6C); the morphology and dimensions of the virus appeared normal. Serial dilutions of MVA-Luc recombinant virus were introduced into 2 × 10^4^ BHK-21 cells cultured in 96-well plates. Cytopathic effects and luciferase expression were assessed at 24 h post-infection. Additionally, BHK-21 cells were infected at an MOI of 0.1, and luciferase expression was measured 1, 6, 12, 24, 48, and 72 h post-infection (Figure 6D). The expression of the luciferase gene in BHK-21 cells was consistent and demonstrated variability depending on the MOI and culture duration.

### 3.8 *In vivo* imaging of luciferase expression after inoculation of MVA-Luc-eGFP

To assess the exogenous gene expression levels of recombinant MVA poxvirus vectors in healthy organisms, we used MVA-Luc-eGFP recombinant viruses at a dose of 5 × 10^7^ IFUs. They were administered to BALB/c mice via intramuscular injection at the lateral aspect of the hind limb muscle and subcutaneous injection at the nape of the neck. This approach enabled us to investigate the *in vivo* distribution and infection levels of the recombinant viruses post-immunization (Figure 6E). One hour after administration, a substantial level of luciferase activity was observed at the injection site in most mice, reaching its peak at 12 h post-infection. This was followed by a gradual decrease, and the signal became undetectable after 72 h.

### 3.9 MVA-Luc-eGFP for screening anti-poxvirus compounds

MVA-Luc-eGFP was used to infect BHK-21 cells for 6 h, after which 200 nM/mL was added to the culture system. Luciferase signal was assessed after 24 h of culture. The rate of luciferase signal inhibition by the compounds was calculated relative to that of DMSO. Tecovirimat, adefovir dipivoxil, and fialuridine inhibited MVA by > 90% (Figure 6F). Furthermore, a stoichiometric relationship was identified between the inhibition rate of these compounds on the Luc signal value and their concentrations when MVA infected BHK-21 cells were treated with varying concentrations of the three compounds.

### 3.10 Construction of MVA-HA

**FIGURE 7:**
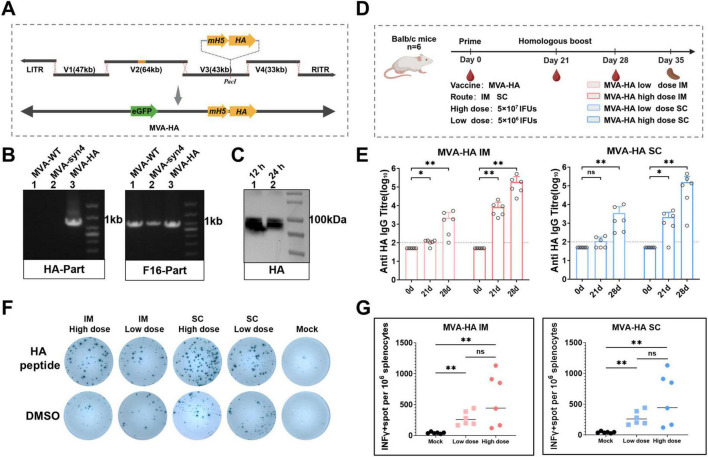
Construction, characterization and Immunological evaluation of MVA-HA. **(A)** The mH5-HA gene expression cassette was incorporated into the V3 plasmid using Gibson assembly. **(B)** Identification of MVA-HA recombinant viruses via PCR analysis. **(C)** Western blot analysis of HA proteins. Protein extracts were obtained from BHK-21 cells infected with MVA-HA at 12 and 24 h post-infection. **(D)** The shcedule of immunization and challenge periods in days, immunizations were performed intramuscularly and subcutaneously. **(E)** IgG binding titres against HA were assessed on day 0, 21 and 28. The crossbars represented the geometric mean of the group ± geometric standard deviation (*n* = 6/group). **(F)** Representative images of IFN-γ ELISpot assay plate wells, where each column represents spleen cells from a mouse and each row represents the indicated stimulus. **(G)** Quantification of antigen-specific IFN-γ-producing T cells. Significance of results were evaluated by unpaired *t*-tests between two groups. *p*-value less than 0.05 was considered significant. *p < 0.05; **p < 0.01; ns., not significant.

We prepared an mH5-HA antigen expression frame DNA fragment and digested the V3 plasmid with *Pac*I. The antigen expression frame DNA fragment, which included homologous regions flanking the *Pac*I digestion site of the V3 plasmid, was ligated into the V3 plasmid using Gibson assembly (Figure 7A). The MVA-HA vaccine was packaged using the MVA-syn packaging system. PCR confirmed that the recombinant virus MVA-HA contained the HA fragment (Figure 7B). Additionally, western blotting analysis demonstrated that MVA-HA expressed antigenic proteins normally after infecting the BHK-21 cells for 12 and 24 h (Figure 7C).

### 3.11 Immunological assessment of MVA-HA

To assess the immunogenicity of the MVA-HA vector vaccine, BALB/c mice were subjected to two homologous immunizations with the sucrose-purified MVA-HA vaccine (Figure 7D). The specific humoral and cellular immune responses elicited by MVA-HA were subsequently evaluated, with the findings presented in Figure 7E. In the low-dose group, significant immune responses were observed following the booster immunization. In contrast, the high-dose group exhibited significant humoral immune responses after the primary and booster immunizations. Additionally, the low-dose group elicited higher levels of cellular immunity than the mock group after intramuscular and subcutaneous booster immunizations (Figures 7F, G).

## 4 Discussion

Poxvirus vectors possess large genomes that can accommodate exogenous gene insertions of up to 25 kb (Smith and Moss, 1983). Genetic engineering approaches for poxviruses primarily involve transfection of homologous DNA segments into cells infected with poxviruses to facilitate homologous recombination (Conrad and Liu, 2019). Subsequent screening of recombinant viruses requires complex plaque purification processes. In this study, we developed a five-plasmid system for packaging a recombinant MVA-associated vaccine with high efficiency. The rescued recombinant MVA exhibited growth characteristics, host range, plaque size, physical properties, and immunogenicity consistent with those of the wild-type virus strain, indicating its potential for the development of chimeric or recombinant poxvirus-based vaccines.

The intricate hairpin structure located at the termini of ITRs in the MVA is crucial for replication processes (Moss, 2013). The MVA terminal hairpin loops consist of 165 residues, which are notably larger than the typical size of approximately 100 bp. Within this loop, there is a perfect tandem repeat of 39 nucleotides located at positions 80–118 and 119–156 (Antoine et al., 1998). During the replication process of MVA, a transient head-to-tail configuration is formed, and this intermediate structure is resolved upon completion of replication (Baroudy et al., 1983). We introduced an innovative approach to address the hairpin loop structure of MVA poxvirus vectors by linking the 9.8-kb-long L-ITR and R-ITR into a continuous loop. The ITR loop combined with other four fragments covering the MVA genome can package the recombinant virus within 3 days in FPV-infected cells, showing great efficiency in poxvirus rescue.

A suitable vector system with a high packaging efficiency is crucial for the development of poxvirus-associated vaccines. With the development of synthetic biology techniques, other teams have reported the rescue of poxviruses by artificial synthesis of the poxvirus genome, which differs from the methods described in this study. In Noyce et al. (2018) conducted a comprehensive study detailing the construction of a synthetic horsepox virus from 10 chemically synthesized DNA fragments. However, for the virus to be rescued, homologous recombination must occur simultaneously among the 10 synthetic fragments, which is inconvenient for recombination operations, and packaging efficiency is not yet clear. In a study conducted by Chiuppesi et al. (2020), the MVA genome was segmented into three subgenomic fragments and MVA poxviruses were packaged using two FPV strains to develop a multi-antigenic severe acute respiratory syndrome coronavirus 2 vaccine candidate. However, the divided poxvirus genome fragments are still larger than 60 kb and bacterial homologous recombination methods are required for genetic manipulation. The gene editing process remains complex, especially when multipoint editing is necessary. In contrast, the division of MVA poxviruses into five subgenomic segments in this study allowed the use of four plasmids, independent of ITRs, for the insertion of exogenous proteins, and *in vitro* recombination methods allowed for convenient artificial editing of the genome, significantly reducing the difficulty of genome editing while ensuring high packaging efficiency. This methodology provides a foundational framework for reverse genetics of other poxvirus species.

The rescued MVA-syn in our study showed similar viral characteristics and immunogenicity to wild-type MVA and was proven to be suitable for the development of an orthopoxvirus vaccine. Orthopoxviruses exhibit a high degree of homology and good cross-reactivity. The MVA vaccine protects mice against severe respiratory infections caused by cowpox virus or VACV when administered intramuscularly or subcutaneously and humoral immune responses, but not cellular immune responses, are necessary and sufficient to mediate anti-MPXV protection (Coulibaly et al., 2005; Drexler et al., 2003). The cross-protective effect was further demonstrated by the observed cross-reactivity with VACV in our experiments and protection against elevated doses of ECTV.

Edghill-Smith et al. (2005) demonstrated that antibody responses elicited by the smallpox vaccine were crucial for preventing fatal monkeypox disease. A recent study by Mucker et al. (2023) reported that the MVA vaccine exhibited a robust immunoprotective effect, offering complete protection against intravenous challenge with MPXV. However, it did not completely alleviate the adverse symptoms of MPXV infection, such as skin tenting and mucosal changes. The results of the present study suggest that higher levels of humoral immunity induced by MVA-syn and MVA-WT provide greater protection in an ECTV challenge model. This model represents a highly virulent infectious disease in mice that is capable of causing mortality even at low doses (Yang et al., 2024). In this study, we conducted ECTV challenge protection experiments to monitor changes in body weight and the occurrence of fur in mice during the challenge phase. Our findings indicated that mice in the high-dose cohort exhibited reduced weight loss, accelerated recovery, and a lower incidence of dull fur irrespective of the administration route (intramuscular or subcutaneous). MVA (Trademark JYNNEOS) developed by Bavarian Nordic A/S has been approved for the prevention of smallpox and monkeypox disease in adults ≥ 18 years of age by the USFDA, and the approved delivery is via the subcutaneous injection. However, intramuscular injection elicited a more robust humoral immune response than subcutaneous injection at equivalent doses, and mice receiving subcutaneous injections exhibited a higher mortality rate and more severe adverse symptoms in the challenge model. Further in-depth delivery studies should be conducted on MVA vaccines in humans.

Consequently, MVA is considered a highly promising vaccine vector for several infectious diseases. In this study, the five-plasmid system for MVA rescue enabled simultaneous insertion of multiple sites, significantly reducing the time required to construct MVA poxvirus-vectored vaccines. The recombinant virus MVA-HA was genetically engineered by incorporating an mH5-HA expression cassette into the V3 plasmid. MVA-HA elicited humoral immune responses in mice after initial and secondary immunizations. Furthermore, robust cellular immunity was observed after the booster immunization, underscoring the immunological efficacy of MVA as a viral vector. The MVA poxvirus vector construction system employed in this study serves as an efficient platform for the rapid development of vaccines, enabling the swift design and production of vaccines targeting emerging pathogens.

In conclusion, we constructed a five-plasmid system that facilitates packaging of the MVA virus, and the synthetic system significantly streamlines the process of creating recombinant poxviruses, offering critical technical insights for the development of recombinant/chimeric poxvirus-vectored vaccines.

## Data Availability

The original contributions presented in this study are included in this article/Supplementary material, further inquiries can be directed to the corresponding authors.
