# Supplementary material for: Design and construction of a fast synthetic modified vaccinia virus Ankara reverse genetics system for advancing vaccine development
Source: Front Microbiol. 2025 Apr 25;16:1572706. doi: 10.3389/fmicb.2025.1572706 (PMC12061933; doi:10.3389/fmicb.2025.1572706)
Supplement: Supplementary file 1 [file Data_Sheet_1.docx]

Supporting Information for

**Design and Construction of a Fast Synthetic Modified Vaccinia Virus Ankara Reverse Genetics System for Advancing Vaccine Development**

**Zhiqiang Gao^1, #,^ Busen Wang^1, #, *^, Tianyu Liu^1^, Zhenghao Zhao^1^, Jinghan Xu^1^, Xiaofan Zhao^1^, Zhe Zhang^1^, Zuyuan Jia^1^, Yilong Yang^1^, Shipo Wu^1^, Wei Chen^1^, Lihua Hou^1, *^**

^1^Laboratory of Advanced Biotechnology, Beijing Institute of Biotechnology, Beijing, People’s Republic of China

^#^These authors contributed equally

^*^Correspondence: [sen154034@163.com (B.S.)](mailto:sen154034@163.com), [houlihua@sina.com](mailto:houlihua@sina.com) (L.H.)


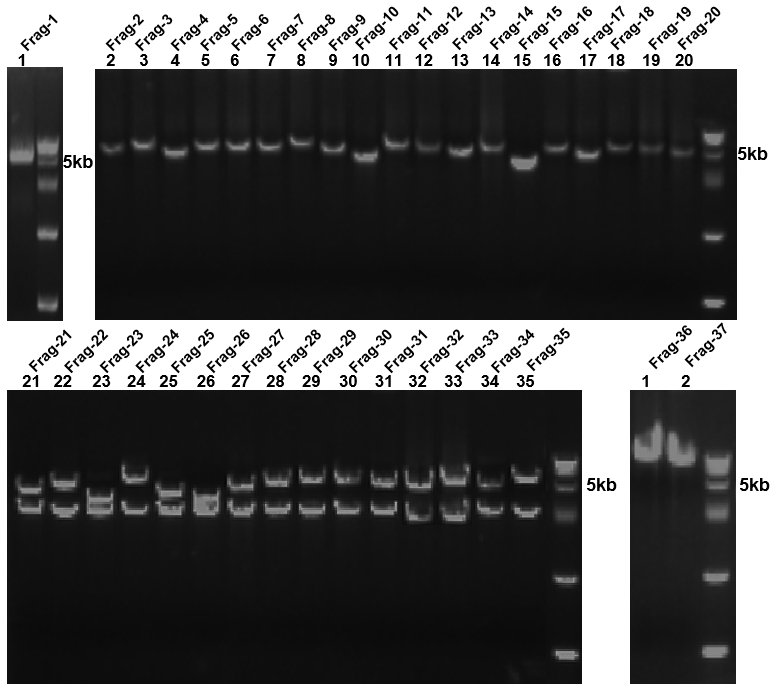


**Figure S1. PCR and digestion results of F1-37**
