# Supplementary material for: Design and construction of a fast synthetic modified vaccinia virus Ankara reverse genetics system for advancing vaccine development
Source: Front Microbiol. 2025 Apr 25;16:1572706. doi: 10.3389/fmicb.2025.1572706 (PMC12061933; doi:10.3389/fmicb.2025.1572706)
Supplement: Supplementary file 2 [file Data_Sheet_2.docx]

Supporting Information for

**Design and Construction of a Fast Synthetic Modified Vaccinia Virus Ankara Reverse Genetics System for Advancing Vaccine Development**

**Zhiqiang Gao^1, #,^ Busen Wang^1, #, *^, Tianyu Liu^1^, Zhenghao Zhao^1^, Jinghan Xu^1^, Xiaofan Zhao^1^, Zhe Zhang^1^, Zuyuan Jia^1^, Yilong Yang^1^, Shipo Wu^1^, Wei Chen^1^, Lihua Hou^1, *^**

^1^Laboratory of Advanced Biotechnology, Beijing Institute of Biotechnology, Beijing, People’s Republic of China

^#^These authors contributed equally

^*^Correspondence: [sen154034@163.com (B.S.)](mailto:sen154034@163.com), [houlihua@sina.com](mailto:houlihua@sina.com) (L.H.)

**Table S1. Primers Used for Amplification of synthetic DNA Fragments**

| **Name** | **Sequence** | **PCR product size (bp)** |
| --- | --- | --- |
| F1-F | CTCTTCCAGAGACGATAGCTG | 5000 |
| F1-R | ATTATATTAAGTCATCTCATATAGATATAGACATCG |  |
| F2-F | ATATGAATAAGCCGTGTTATC | 5000 |
| F2-R | TATAGAGTGTACTCGTTAC |  |
| F3-F | CGTTGTTTAAGTTGGATG | 5209 |
| F3-R | ATATGATCATCAAACGAACTG |  |
| F4-F | CATTGTCCAGATTGAACATAC | 4274 |
| F4-R | GATAACTGAAAAAAAATTTATTGTTATTG |  |
| F5-F | GGATTGGGAAAATAACAAATC | 4941 |
| F5-R | AAAAGTGAAAAACAATATTATTTTTATCGTTGGTTGTTAC |  |
| F6-F | CCTCTCTTCAACATCTTTGATCTC | 4886 |
| F6-R | GCATAGAAAAAAAACAAAATGAAATTCTAC |  |
| F7-F | CAATCACCACTTTCATATTTAGA | 4785 |
| F7-R | AAAAAATGATAAAATAAATTAGTTTTATTGCTGGTTGTG |  |
| F8-F | GCGTCAGAACGCTCGTCAATATAGATC | 5294 |
| F8-R | GCATAAAATAATTTACAACAGTTGTACG |  |
| F9-F | CTACAAAAATTACTCCAGCCG | 4541 |
| F9-R | GTGCTCTGACACTATCAGCCATC |  |
| F10-F | GACATTCCTATGATGATGCAG | 3877 |
| F10-R | GGTTAAACAAAAACATTTTTATTCTCAAATGAG |  |
| F11-F | GATTACCTTACTCATGATTAAACCTAAATAATTGTAC | 5120 |
| F11-R | CCATGATAAATGGAAAGATATACAG |  |
| F12-F | TATATATGACATAATAAATTGGTAAATCCTAGTTCTGG | 4861 |
| F12-R | CCGGTTACCGAATAAAATATCTTCCTGAGTATTTT |  |
| F13-F | GAACTAGTCGATCCAATAAATTATCAAATACTTC | 4390 |
| F13-R | GCCACCCATATTTATCTCATTGAAAG |  |
| F14-F | GGAATGTTGTTTTCGGATAGAGTTC | 5081 |
| F14-R | CAACAATGTCTGGAAAGAACTGTCC |  |
| F15-F | GGATGTGCTGCAAGGCG | 3510 |
| F15-R | CAGCCATTTTTATCAAGTCAGTTTC |  |
| F16-F | CTATTATTTTATAGTTGTAATAAAAAGGGAAATTTG | 5035 |
| F16-R | CTATTCCATATTACTAAAATCGGAACACC |  |
| F17-F | GAGATGTTAAATGAATTAGGTTATAGCGG | 4245 |
| F17-R | CGCATTTACTTCTTACAAGTTTTAAC |  |
| F18-F | GCTATTACAACACTATCTGATCTAGTCACCG | 5186 |
| F18-R | TCACTGAATTCATTATATCAAATTAGATACC |  |
| F19-F | TTGTATTCGCTTGGTTTAACGGC | 5106 |
| F19-R | TTTATTTCTTTATATATCTCATCAGTTTTATGGAG |  |
| F20-F | TTGTTTGGGGAGTTAACTTTCGTAAAG | 4799 |
| F20-R | ATTTTTAAAACATAGTTATTACTTATCACTCATAAATGAG |  |
| PET-F | GCACGAAGTTCTTCGGCGGTTGGCGCGCCGCACCATTATGTTCCGGATCTG | 2542 |
| PET-R | GATTGCACGAAGTTCTTCGGCGGTTGCGATCGCTGTGCGCGGAACCCCTATTTG |  |

**Table S2. Restriction enzyme Used for Digestion of synthetic DNA Fragments**

| **Name** | **Cleavage by restriction enzyme** | **Fragment** | **Digest product (bp)** |
| --- | --- | --- | --- |
| PUC57-F21 | *AsisI* | F21 | 4042 |
| PUC57-F22 | *AsisI* | F22 | 4556 |
| PUC57-F23 | *AsisI* | F23 | 3410 |
| PUC57-F24 | *AsisI* | F24 | 5075 |
| PUC57-F25 | *AsisI* | F25 | 3806 |
| PUC57-F26 | *AsisI* | F26 | 3395 |
| PUC57-F27 | *AsisI* | F27 | 4338 |
| PUC57-F28 | *AsisI* | F28 | 4724 |
| PUC57-F29 | *AsisI* | F29 | 5033 |
| PUC57-F30 | *AsisI* | F30 | 5000 |
| PUC57-F31 | *AsisI* | F31 | 4755 |
| PUC57-F32 | *AsisI* | F32 | 4347 |
| PUC57-F33 | *AsisI* | F33 | 4855 |
| PUC57-F34 | *AsisI* | F34 | 4669 |
| PUC57-F35 | *AsisI* | F35 | 5393 |
| PUC57-F36 | *AsisI + XmaI* | F36 | 9640 |
| PUC57-F37 | *AsisI + XmaI* | F37 | 9794 |

**Table S3. Stitching oligonucleotides for TAR**

| **Name** | **Stitching oligonucleotides** | **Length（bp）** |
| --- | --- | --- |
| PYE-Link-F1-F | TGCGTCCCTGTTTGCATTATGAATTAGTTACGCTAGGGATAACAGGGTAATATAGAACCCGAACGACCGAGCGCAGCGGCGCGATCGCCTCTTCCAGAGACGATAGCTGATTGAGTACAAAGTCCAATGATTGCACGAAGTTCTTCGGCGGTTTTCATGGAGTCATTT | 168 |
| PYE-Link-F1-R | AAATGACTCCATGAAAACCGCCGAAGAACTTCGTGCAATCATTGGACTTTGTACTCAATCAGCTATCGTCTCTGGAAGAGGCGATCGCGCCGCTGCGCTCGGTCGTTCGGGTTCTATATTACCCTGTTATCCCTAGCGTAACTAATTCATAATGCAAACAGGGACGCA | 168 |
| F10-Link-PYE-F | TAAACCTAAATAATTGTACTTTGTAATATAATGATATATATTTTCACTTTATCTCATTTGAGAATAAAAATGTTTTTGTTGGCGCGCCATTAAAGTCAGTGAGCGAGGAAGCGCGTAACTATAACGGTCCTAAGGTAGCGAATCCTGATGCGGTATTTTCTCCTTACG | 168 |
| F10-Link-PYE-R | CGTAAGGAGAAAATACCGCATCAGGATTCGCTACCTTAGGACCGTTATAGTTACGCGCTTCCTCGCTCACTGACTTTAATGGCGCGCCAACAAAAACATTTTTATTCTCAAATGAGATAAAGTGAAAATATATATCATTATATTACAAAGTACAATTATTTAGGTTTA | 168 |
| F21-Link-F10-F | GCACATGCAGAAGAACACCGCTTAGGCGGATTCAGTACAATGTTATATTTTTCGTACCAACTCATTTAAATATCATAATCGGCGCGCCTTCCTATGATGATGCAGAATTTTGGATAACACGGTATTGATGGTATCTGTTACCATAATTCCTTTGATGGCTGATAGTGT | 168 |
| F21-LinkF10-R | ACACTATCAGCCATCAAAGGAATTATGGTAACAGATACCATCAATACCGTGTTATCCAAAATTCTGCATCATCATAGGAAGGCGCGCCGATTATGATATTTAAATGAGTTGGTACGAAAAATATAACATTGTACTGAATCCGCCTAAGCGGTGTTCTTCTGCATGTGC | 168 |
| F14-Link-PYE-F | ACTATGTGATGTCTTGGAATCAATTACAGATTTCTCCGTGATAGGTATCGATGAAGGACAGTTCTTTCCAGACATTGTTGATTAAAGTCAGTGAGCGAGGAAGCGCGTAACTATAACGGTCCTAAGGTAGCGAATCCTGATGCGGTATTTTCTCCTTACG | 160 |
| F14-Link-PYE-R | CGTAAGGAGAAAATACCGCATCAGGATTCGCTACCTTAGGACCGTTATAGTTACGCGCTTCCTCGCTCACTGACTTTAATCAACAATGTCTGGAAAGAACTGTCCTTCATCGATACCTATCACGGAGAAATCTGTAATTGATTCCAAGACATCACATAGT | 160 |
| PYE-Link-F15-F | TGCGTCCCTGTTTGCATTATGAATTAGTTACGCTAGGGATAACAGGGTAATATAGAACCCGAACGACCGAGCGCAGCGGCGCGATCGCGGATGTGCTGCAAGGCGATTAAGTTGGGTAACGCCAGGGTTTTCCCAGTCACGACGTTGTAAAACGACGGCCAGTGAGCG | 168 |
| PYE-Link-F15-R | CGCTCACTGGCCGTCGTTTTACAACGTCGTGACTGGGAAAACCCTGGCGTTACCCAACTTAATCGCCTTGCAGCACATCCGCGATCGCGCCGCTGCGCTCGGTCGTTCGGGTTCTATATTACCCTGTTATCCCTAGCGTAACTAATTCATAATGCAAACAGGGACGCA | 168 |
| PYE-Link-F21-F | TGCGTCCCTGTTTGCATTATGAATTAGTTACGCTAGGGATAACAGGGTAATATAGAACCCGAACGACCGAGCGCAGCGGCGCGATCGCCGATATAGGCCGCGTGTGATTTACTCATTTATGAGTGATAAGTAATAACTATGTTTTAAAAATCACAGCAGTAGTTTAAC | 168 |
| PYE-Link-F21-R | GTTAAACTACTGCTGTGATTTTTAAAACATAGTTATTACTTATCACTCATAAATGAGTAAATCACACGCGGCCTATATCGGCGATCGCGCCGCTGCGCTCGGTCGTTCGGGTTCTATATTACCCTGTTATCCCTAGCGTAACTAATTCATAATGCAAACAGGGACGCA | 168 |
| F30-Link-PYE-F | TGCGTCCCTGTTTGCATTATGAATTAGTTACGCTAGGGATAACAGGGTAATATAGAACCCGAACGACCGAGCGCAGCGGCGCGATCGCCGATATAGGCCGCGTGTGATTTACTCATTTATGAGTGATAAGTAATAACTATGTTTTAAAAATCACAGCAGTAGTTTAAC | 168 |
| F30-Link-PYE-R | GTTAAACTACTGCTGTGATTTTTAAAACATAGTTATTACTTATCACTCATAAATGAGTAAATCACACGCGGCCTATATCGGCGATCGCGCCGCTGCGCTCGGTCGTTCGGGTTCTATATTACCCTGTTATCCCTAGCGTAACTAATTCATAATGCAAACAGGGACGCA | 168 |
| PYE-Link-F29-F | TGCGTCCCTGTTTGCATTATGAATTAGTTACGCTAGGGATAACAGGGTAATATAGAACCCGAACGACCGAGCGCAGCGGCGCGATCGCATTCTGATGTATCACAAGAAGTTAGAAAGTATTTTTGTGTTAAAACAATGAACTAATATTTATTTTTGTACATTAATAAA | 168 |
| PYE-Link-F29-R | TTTATTAATGTACAAAAATAAATATTAGTTCATTGTTTTAACACAAAAATACTTTCTAACTTCTTGTGATACATCAGAATGCGATCGCGCCGCTGCGCTCGGTCGTTCGGGTTCTATATTACCCTGTTATCCCTAGCGTAACTAATTCATAATGCAAACAGGGACGCA | 168 |
| F35-Link-PYE-F | AATGACTCCATGAAAACCGCCGAAGAACTTCGTGCAATCATTGGACTTTGTACTCAATCAGCTATCGTCTCTGGAAGAGTGGCGCGCCATTAAAGTCAGTGAGCGAGGAAGCGCGTAACTATAACGGTCCTAAGGTAGCGAATCCTGATGCGGTATTTTCTCCTTACG | 168 |
| F35-Link-PYE-R | CGTAAGGAGAAAATACCGCATCAGGATTCGCTACCTTAGGACCGTTATAGTTACGCGCTTCCTCGCTCACTGACTTTAATGGCGCGCCACTCTTCCAGAGACGATAGCTGATTGAGTACAAAGTCCAATGATTGCACGAAGTTCTTCGGCGGTTTTCATGGAGTCATT | 168 |

**Table S4. Primers Used for Identification of the MVA-syn genome**

| **Name** | **Sequence** | **Product size(bp)** |
| --- | --- | --- |
| BACYAC-Part-F | GGCGTCAGTCCACCAGC | 754.00 |
| BACYAC-Part-R | GACGCCAGATGGCAGTAG |  |
| F1-Part-F | GCGGTCCAGAGGGAGATG | 510.00 |
| F1-Part-R | GGTGATACGGAACCACCC |  |
| F6-Part-F | CGAATGGTCGGCTCTCTG | 706.00 |
| F6-Part-R | GGGAGATATAGCACTGGC |  |
| F16-Part-F | CGGGACACGCTCTTAGG | 553.00 |
| F16-Part-R | GATAGATGTACTGGGACC |  |
| F26-Part-F | GGGAGAACTTAGGCGGC | 752.00 |
| F26-Part-R | CCCTAACGGAGTTATCC |  |
| F30-Part-F | GTCTGAGGTGTTCGTCTAG | 610.00 |
| F30-Part-R | GTGCGGTATGAGTTCTCGC |  |
| FPV-Part-F | GTATCCTTGTTATCATCGC | 681.00 |
| FPV-Part-R | AGGAGGTAGCGGATCAGG |  |
| RITR-Part-F | GTCTCCGAATGCGGCATG | 689.00 |
| RITR-Part-R | CCGGCATCATAAACACGG |  |
| HA-Part-F | CACAACCGCCAACGCCG | 617.00 |
| HA-Part-R | CAAACACGTAGGCATCGGC |  |
